# *In Vitro* Effects of Low-Intensity Pulsed Ultrasound Stimulation on the Osteogenic Differentiation of Human Alveolar Bone-Derived Mesenchymal Stem Cells for Tooth Tissue Engineering

**DOI:** 10.1155/2013/269724

**Published:** 2013-09-30

**Authors:** KiTaek Lim, Jangho Kim, Hoon Seonwoo, Soo Hyun Park, Pill-Hoon Choung, Jong Hoon Chung

**Affiliations:** ^1^Department of Biosystems & Biomaterials Science and Engineering, Seoul National University, Seoul 151-921, Republic of Korea; ^2^Department of Oral and Maxillofacial Surgery and Dental Research Institute, School of Dentistry, Seoul National University, Seoul, Republic of Korea; ^3^Tooth Bioengineering National Research Laboratory of Post BK21, School of Dentistry, Seoul National University, Seoul, Republic of Korea; ^4^Research Institute for Agriculture and Life Sciences, Seoul National University, Seoul 151- 921, Republic of Korea

## Abstract

Ultrasound stimulation produces significant multifunctional effects that are directly relevant to alveolar bone formation, which is necessary for periodontal healing and regeneration. We focused to find out effects of specific duty cycles and the percentage of time that ultrasound is being generated over one on/off pulse period, under ultrasound stimulation. Low-intensity pulsed ultrasound ((LIPUS) 1 MHz) with duty cycles of 20% and 50% was used in this study, and human alveolar bone-derived mesenchymal stem cells (hABMSCs) were treated with an intensity of 50 mW/cm^2^ and exposure time of 10 min/day. hABMSCs exposed at duty cycles of 20% and 50% had similar cell viability (O.D.), which was higher (*P < 0.05) than that of control cells. The alkaline phosphatase (ALP) was significantly enhanced at 1 week with LIPUS treatment in osteogenic cultures as compared to control. Gene expressions showed significantly higher expression levels of CD29, CD44, COL1, and OCN in the hABMSCs under LIPUS treatment when compared to control after two weeks of treatment. The effects were partially controlled by LIPUS treatment, indicating that modulation of osteogenesis in hABMSCs was related to the specific stimulation. Furthermore, mineralized nodule formation was markedly increased after LIPUS treatment than that seen in untreated cells. Through simple staining methods such as Alizarin red and von Kossa staining, calcium deposits generated their highest levels at about 3 weeks. These results suggest that LIPUS could enhance the cell viability and osteogenic differentiation of hABMSCs, and could be part of effective treatment methods for clinical applications.

## 1. Introduction 

Many research studies have been conducted on cell proliferation and differentiation using ultrasound stimulators, as well as the development of therapeutic applications. In addition, commercially available clinical products using this technology have already been released. Ultrasound stimulation is acoustic energy at frequencies above the limit of human hearing. It is a form of mechanical energy that can be conducted into the body as high-frequency acoustical waves. The micromechanical strains produced by these pressure waves in body tissue can result in biochemical events at the cellular level [1–3]. 

*In vitro* studies have suggested that LIPUS treatment produces significant multifunctional effects that are directly relevant to bone formation and resorption. Clinical investigations involving LIPUS have shown successful healing of delayed unions and nonunions. LIPUS has been widely found to stimulate fracture healing in animal models and in clinical treatments [4, 5]. LIPUS has also been reported to accelerate bone maturation in distraction osteogenesis cases in animal models [6, 7] and in clinical treatments [8, 9]. LIPUS may induce a micromechanical stimulation of the bone and induce osteogenesis, according to Wolff's Law [10]. In particular, the differential absorption of LIPUS may establish a gradient of mechanical strain in the healing callus that stimulates periosteal bone formation [11, 12].

However, the exact use of ultrasound stimulators has been controversial due to side effects related to proper intensities or time, as well as other parameter choices such as duty cycle. Thus, we sought to provide further guidance to the use of LIPUS by evaluating the effects of duty cycles of 20% and 50% during 10 min per day. Our research team has already investigated and reported on the effects of LIPUS on proliferation and differentiation of hABMSCs across a range of intensities of ultrasonic power [13]. We ascertained that LIPUS treatment was effective in promoting the proliferation and osteogenic differentiation of hABMSCs. 

However, these and other preliminary findings regarding LIPUS did not investigate the effects of changes in the duty cycle of the ultrasound stimulators. The role of the duty cycle is particularly important because of the method delivered to tissues during peak operation times. There are no previous studies investigating the effects of the low duty cycle condition of the LIPUS treatment on the cell growth and differentiation of hABMSCs. In addition, despite its pronounced effects during the osteogenesis process, the underlying mechanism of LIPUS remains unclear.

Thus, this study examines the effects of LIPUS treatments with differing pulsed duty cycles on *in vitro* cell growth and osteogenic differentiation of hABMSCs. The aim of this study was to investigate the effects of LIPUS (with duty cycles of 20% and 50%) on proliferation and differentiation of hABMSCs for tooth tissue engineering.

## 2. Materials and Methods

### 2.1. Cell Culture

hABMSCs were taken from the Intellectual Biointerface Engineering Center, Dental Research Institute, College of Dentistry, and Seoul National University. The cells were cultured in alpha-minimum essential medium ((*α*-MEM) Welgene Inc., Korea) supplemented with 10% fetal bovine serum ((FBS) Welgene Inc., Korea), 10 mM L-ascorbic acids (Sigma, USA), and antibiotics (10,000 U/mL penicillin, 10 mg/mL streptomycin, and 25 ug/mL amphotericin B). hABMSCs were placed in 100 mm culture dishes at a density of 3.0 × 10^4^ cells/cm^2^. Cells were maintained in a humidified incubator at 37°C and 5% CO_2_. Medium was replaced every 2-3 days. After reaching more than 70% confluence, the cells were cultured for about 2-3 weeks in induction media for osteogenic differentiation, which was prepared with *α*-MEM, 10 mM L-ascorbic acids, 10% FBS, antibiotics, 10 mM *β*-glycerophosphate, and 100 nM dexamethasone (Sigma, USA). Osteogenic medium was changed once every 2-3 days. Passage 3–5 cells were used for our studies.

### 2.2. LIPUS Treatment

**Figure 1:**
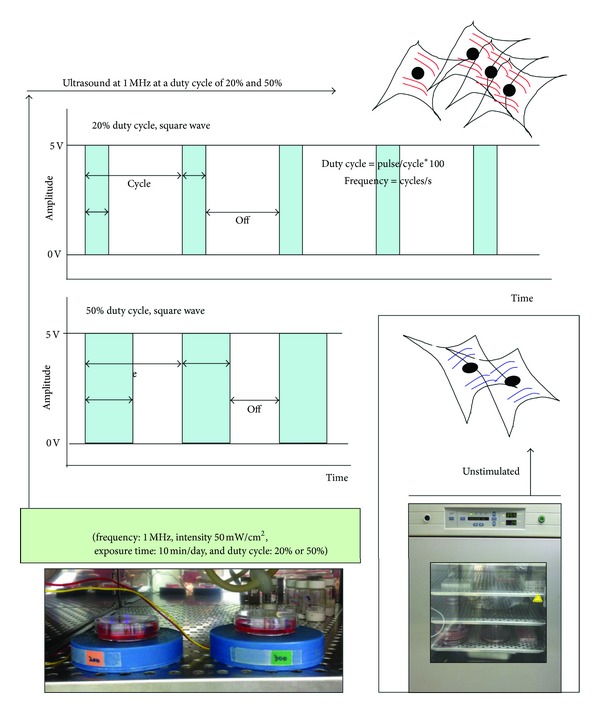
Schematic diagram of LIPUS treatment (frequency: 1 MHz, intensity: 50 mW/cm^2^, exposure time: 10 min/day, and duty cycle: 20% or 50%), as compared to static culture as control.

hABMSCs were placed into 35 mm culture dishes at an initial density 1 × 10^4^ cells/well. We carried out with three group conditions as follows: (1) control group (osteogenic differentiation media without LIPUS treatment), (2) osteogenic differentiation media with LIPUS treatment at a 20% duty cycle for 10 min once a day, and (3) osteogenic differentiation media with LIPUS treatment at a 50% duty cycle for 10 min once a day (Figure 1). The hABMSCs were treated with pulsed ultrasound at 1 MHz at duty cycles of 20% and 50% at low intensity of 50 mW/cm^2^. The transducer was sterilized in 70% ethanol. A culture plate was placed above the transducer, and coupling gel (Choongwae Pharma Co., Korea) was covered on the transducer.

### 2.3. Cell Viability, DNA Proliferation, *In Vitro* Migration, and FE-SEM Morphological Analysis

The cell growth of hABMSCs was measured by WST-1 assay (EZ-Cytox Cell Viability Assay Kit, Daeillab Service Co., Ltd.) as manufacture's protocols. The formazan dye produced by viable cells was quantified by a multiwell spectrophotometer (Victor 3, Perkin Elmer, USA), measuring the absorbance of the dye solution at 460 nm. DNA concentration was quantified by fluorometry using the CyQUANT Cell Proliferation Assay Kit (Invitrogen) and the *λ* DNA standard (Invitrogen). The cell proliferation was measured using a Cytofluor II fluorescence multiwell plate reader with excitation of 485 nm and emission of 530 nm according to the instructions of the manufacturer. hABMSCs were cultured with or without LIPUS, and cell morphology was observed by phase contrast microscopy (Nikon TS100, Japan). *In vitro* cell migration was assessed by CytoSelect Wound Healing Assay as manufacture's protocols. Wound closure was measured by microscopy for up to 72 h, and photographs were taken. hABMSCs were stimulated with exposure to LIPUS for 72 h except for the control (without stimulation group). Cell morphologies of hABMSCs were observed by a field-emission scanning electron microscope ((FESEM) JEOL, JSM-5410LV) at 2 kV accelerating voltage.

### 2.4. Measurement of Mineralized Nodule Formation

Alkaline phosphatase (ALP) activity of the cell layer was quantified spectrophotometrically according to the instructions of the Sensolyte ALP Assay kit (AnaSpec, USA). After centrifugation at 2500 ×g for 10 min at 4°C, enzyme activity was calculated by measuring the yellow p-nitrophenol product formed at 405 nm. The cells exposed at induction treatment were exposed to LIPUS for 2-3 weeks (10 min duration/day) except for control. Condition and nodule formation were checked routinely by phase contrast microscopy. Alizarin red is a common histochemical technique used to detect calcium deposits in mineralized tissues and cultures. Briefly, the ethanol-fixed cells and matrix were stained for 1 h with 40 mM Alizarin red-S (pH 4.2) and extensively rinsed with water. After photography, the bound stain was eluted with 10% (wt/vol) cetylpyridinium chloride, and Alizarin red-S in samples was quantified by measuring absorbance at 544 nm. Vitamin C, *β*-glycerophosphate, Alizarin red-S, and cetylpyridinium chloride were obtained from Sigma-Aldrich (St. Louis, MO, USA). hABMSCs were also cultured in osteogenic medium for 2-3 weeks in order to investigate assessment of mineralization using von Kossa staining, with and without LIPUS. Cells were fixed with 4% (wt/vol) formaldehyde in PBS during 15 min. And, the cells were incubated in 5% (wt/vol) silver nitrate (Sigma-Aldrich, USA) for 1 hour on the UV light condition, followed by incubation in 5% (wt/vol) sodium thiosulfate (Sigma-Aldrich, USA) for 5 min. The wells were finally rinsed with DW twice by air-dried, and captured, mineralization images using an optical microscope.

### 2.5. Reverse Transcriptase: Polymerase Chain Reaction Analysis

RT-PCR was used to measure the expression of various osteogenic factors. After 10 days in OSS culture, total RNA was isolated with TRIzol reagent (Invitrogen) and used to synthesize cDNA using a first-strand cDNA synthesis kit (Invitrogen) according to the instructions of the manufacturer. The human primers used in this study are listed in Table 1. RNA was extracted from the cell cultures at 14 days after the addition of differentiation media. These extracts were subjected to RT-PCR analysis with CD29, CD44, COL1, OCN, and GAPDH as the positive control. The products were separated by electrophoresis on a 1% agarose gel (SeaKem ME; FMC Bioproducts) and visualized by ultraviolet-induced fluorescence. Each band was normalized to a housekeeping gene expressed in the same amount in the different samples. Expression levels of gene areas were measured using ImageJ 1.45s (National Institutes of Health). 

### 2.6. Confocal Microscopy and Immunohistochemistry

The cells were washed in phosphate buffered saline ((PBS) Sigma-Aldrich, Milwaukee, WI, USA), fixed in a 4% paraformaldehyde solution (Sigma-Aldrich, Milwaukee, WI, USA) for 20 min, and permeabilized with 0.2% Triton X-100 (Sigma-Aldrich, Milwaukee, WI, USA) for 15 min. Cells were incubated with TRITC conjugated phalloidin, antiosteocalcin, its secondary antibody (Cat. no. AB10911, Millipore), and DAPI (Millipore, Billerica, MA, USA) according to the manufacture's protocol. Cytoskeleton organization was visualized using an actin cytoskeleton and focal adhesion staining kit (FAK100; Millipore, Billerica, MA) according to the manufacturer's instruction. In addition, stem cell surface markers of mesenchymal stem cells were captured using Stro-1 (Santa Cruz Biotechnology, USA) and CD146 (BD Bioscience, USA) according to the manufacturer's instruction. Cells were mounted in glycerol/buffer on a glass slide after extensive washing with PBS. Images of labeled cells were obtained by a Confocal Laser Scanning Microscope (Carl Zeiss, LSM710) and histogram was extracted using MATLAB (R2013a, Mathworks, USA) to investigate the diverse cellular dynamics labeled with fluorescent indicators.

### 2.7. Statistical Analysis

Statistical analysis was carried out using the SAS Statistical Analysis System for Windows v8.2 (SAS Institute, Inc., Cary, NC, USA). Statistical significance between control and treatment groups was compared with two-way ANOVA and Duncan's multiple range tests at *P < 0.05. The data were reported as the mean ± standard deviation.

## 3. Results 

### 3.1. Immunocytochemistry Analysis of hABMSCs for Stem Cell Markers

**Figure 2:**
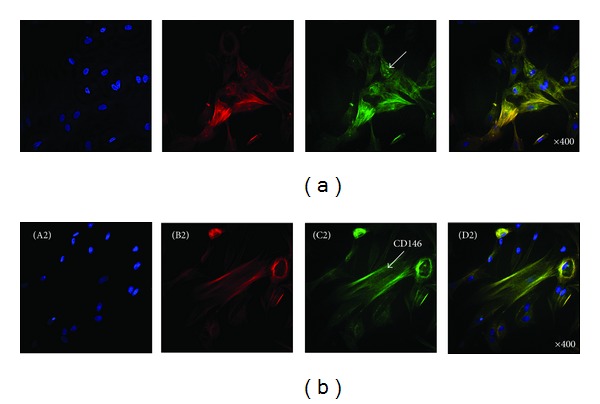
Representative immunocytochemistry images of hABMSCs. Fluorescence images of hABMSCs showed cell nuclei (A1), actin filaments (B1), Stro-1 (C1), and merged images (D1) of the fluorescence stains (a). Fluorescence images of hABMSCs showed cell nuclei (A2), actin filaments (B2), CD146 (C2), and merged images (D2) of the fluorescence stains (b) as MSC markers.

For investigating stem cell characteristics, we measured the cell morphologies of hABMSCs via immunocytochemistry and analyzed positive markers. Representative immunocytochemistry images of hABMSCs are shown in Figure 2(a). Fluorescence images of hABMSCs show cell nuclei (A1), actin filaments (B1), Stro-1 (C1), and merged images (D1) of the fluorescence stains (Figure 2(b)). Fluorescence images of hABMSCs showed cell nuclei (A2), actin filaments (B2), CD146 (C2), and merged images (D2) of the fluorescence stains as MSC markers. Based on the immunocytochemistry analysis, the hABMSCs to be used for the study showed characteristics of mesenchymal stem cells.

### 3.2. Cell Morphology, Cell Viability, and FE-SEM Morphological Analysis

**Figure 3:**
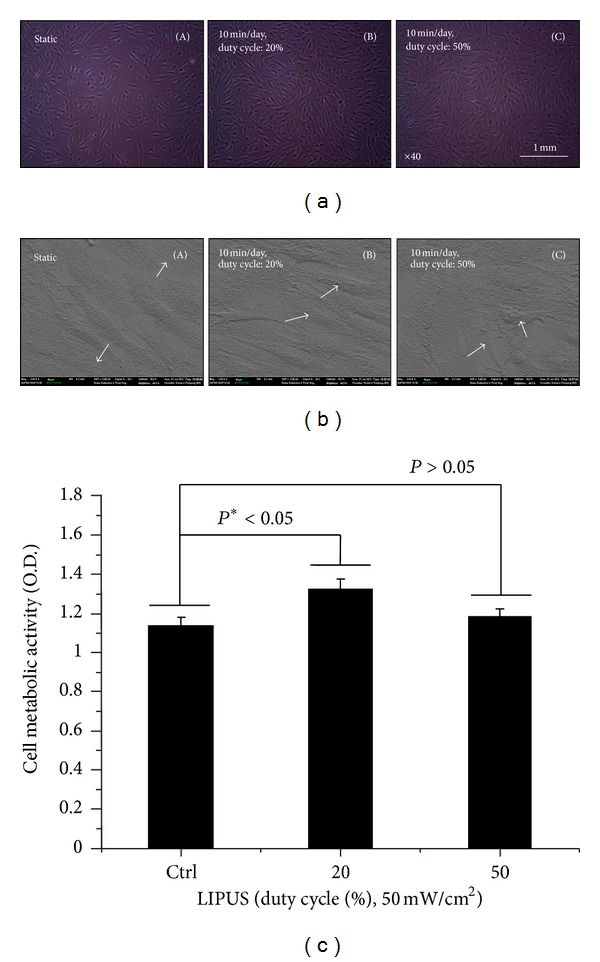
Representative optical microscopic images of hABMSCs stimulated for 4 days in static condition (A), at 20% duty cycle (B), and at 50% duty cycle (C) under LIPUS treatment (a). Representative FE-SEM morphologies of hABMSCs stimulated for 7 days in static condition (A), at 20% duty cycle (B), and 50% duty cycle (C) under LIPUS treatment (b). FE-SEM images showed more lining up observation at stimulation groups compared to control group (arrows: cell direction). Cell metabolic viability as optical density of hABMSCs measured using WST-1 (c). Overhead brackets with asterisks indicate significant difference between groups.

We obtained representative morphologies of hABMSCs for 4 days in static condition (A), at 20% duty cycle under LIPUS (B), and at 50% duty cycle under LIPUS (C) (Figure 3(a)). As shown in the cell images, cells under 20% duty cycle LIPUS for 10 min/day, compared to those in the static culture, had much higher (*P < 0.05) cell density. In addition, Figure 3(b) shows representative FE-SEM cell shape morphologies of hABMSCs cultured for 7 days in static condition (A) at 20% duty cycle under LIPUS (B), and at 50% duty cycle under LIPUS (C) (b). Cell metabolic viability was measured as optical density of hABMSCs using WST-1 (Figure 3(c)). This value indicated not much higher than control group. In addition, we evaluated the difference between the presence (+) or absence (−) of FBS in culture media on effects of LIPUS (Figure S1; see Supplementary Material available online at http://dx.doi.org/10.1155/2013/269724). Figure S1 shows representative optical microscopic images of hABMSCs stimulated for 4 days as follows: Figure S1(A) is FBS (+) proliferation media with cells in static condition (a), at 20% duty cycle under LIPUS (b), and at 50% duty cycle under LIPUS (c). Figure S1(B) is FBS (−) media with cells in static condition (a), at 20% duty cycle under LIPUS (b), and at 50% duty cycle under LIPUS (c). Cell metabolic viability as optical density of hABMSCs between FBS (+) and FBS (−) groups was measured using WST-1 (Figure S1(C)). This showed that LIPUS treatment of ABMSCs has a limit to cell growth, migration, and differentiation under the FBS (−) condition. Our initial expectation was that the cells would be grown and confluent in culture dishes receiving the LIPUS treatment without the addition of FBS (−). Based on these results, we can conclude that the LIPUS treatment supports cell growth or at least give synergic effects to cells. 

### 3.3. Cell Proliferation and *In Vitro* Migration

**Figure 4:**
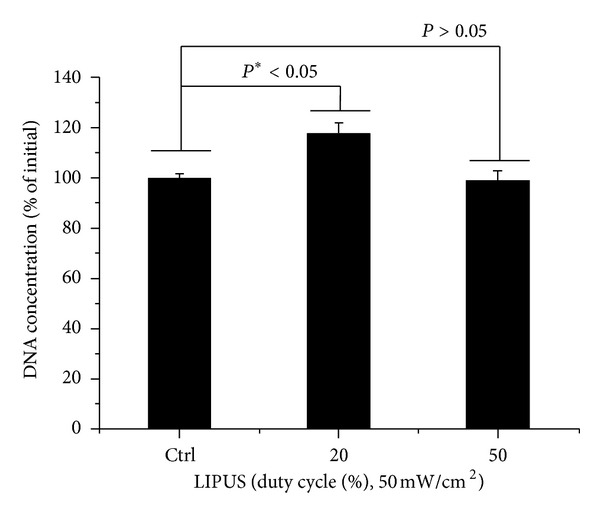
DNA concentration as percent of initial of hABMSCs measured using CyQUANT Cell Proliferation Assay Kit (D) (*n* = 3).

**Figure 5:**
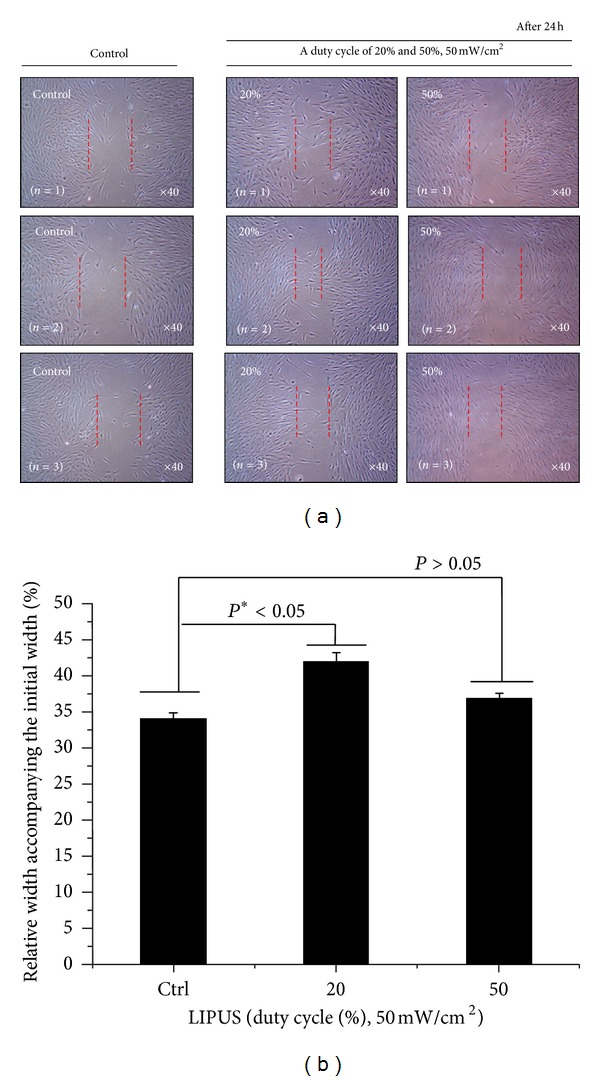
*In vitro* cell migration as representative optical microscopic images of LIPUS group compared to the static culture (a), indicating that the stimulation group exposed at 20% duty cycle LIPUS for 10 min/day was significantly different (*P < 0.05) among groups (b) (*n* = 3).

The proliferation of cells stimulated at 20% duty cycle LIPUS increased by 10% compared to the control (*P < 0.05). As a consequence, both cell viability and cell proliferation were significant at duty cycle of 20% during 10 min/day LIPUS. Results of an *in vitro* migration assay of hABMSCs are shown in Figure 4. *In vitro* cell migration, shown as representative optical microscopic images of the LIPUS group compared to the static culture, shows the stimulation group exposed at 20% duty cycle LIPUS for 10 min/day as significantly different (*P < 0.05) among groups (Figure 5). Exposing hABMSCs to LIPUS forces reveals signs of increased metabolic activity such as ion transportation, fibroblast migration, protein synthesis, and others. One interesting result from our study is that the cells in the lower duty cycle group were more proliferated and differentiated than those in the other groups. Figure S2(A) shows *in vitro* cell migration as representative optical microscopic images with FBS (+) group under LIPUS treatment compared to the static culture. This showed that the cell migration of LIPUS group exposed at 20% duty cycle was faster than 50% duty cycle group (B). Figure S2(B) indicated *in vitro* cell migration as representative optical microscopic images with FBS (−) group under LIPUS treatment compared to static culture. LIPUS treatment with absence (−) of FBS in osteogenic media was ineffective on cell growth and migration. Based on the result, we could ascertain that LIPUS treatment had a synergy effect on cell proliferation and migration when exposing presence of FBS in culture media. 

### 3.4. Gene Expression of Osteoblastic Differentiation Markers

**Figure 6:**
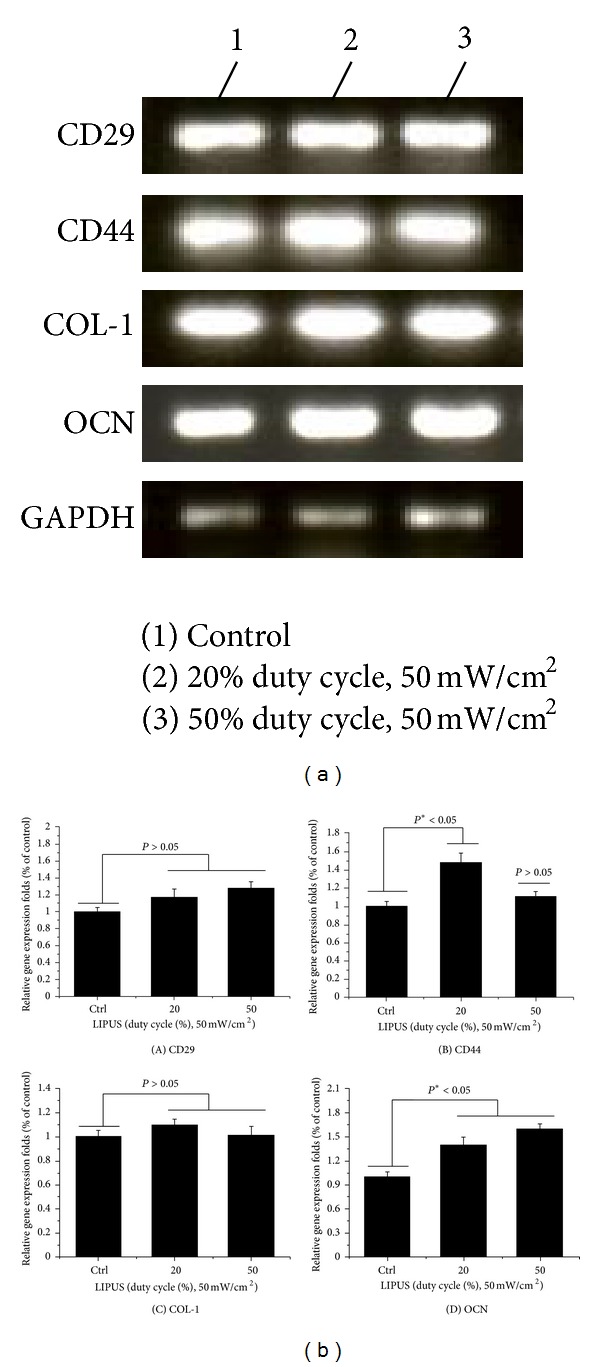
RT-PCR analysis of cell cultures between LIPUS treatment and static culture for 2 weeks. Expression of genes associated with the osteoblastic differentiation was examined using real time PCR to investigate the effect of LIPUS treatment on gene expression (a). Expression levels (b) of CD29 (A), CD44 (B), COL1 (C), and OCN (D) at 2 weeks were significantly higher in LIPUS treatment. Stimulation groups exposed during 10 min/day at 20% duty cycle (in CD44) or 50% duty cycle (in OCN) were significantly different (*P < 0.05) among groups.

The expression of genes associated with osteoblastic differentiation was examined using RT-PCR to investigate the effect of LIPUS on gene expression.  Figure 6(a) shows RT-PCR analysis of the static and stimulated cell cultures after a 2-week period. Expression levels (Figure 6(b)) of CD29 (A), CD44 (B), COL1 (C), and OCN (D) at 2 weeks were significantly higher in LIPUS treatment. Stimulation groups exposed during 10 min/day at 20% duty cycle (for CD44) or 50% duty cycle (for OCN) were significantly different (*P < 0.05) between the groups. Expression levels were measured using ImageJ 1.45s. As a result of these increases in expression, we can say that LIPUS is affiliated with mechanotransduction. 

### 3.5. Enhanced Osteogenic Differentiation of hABMSCs via LIPUS

**Figure 7:**
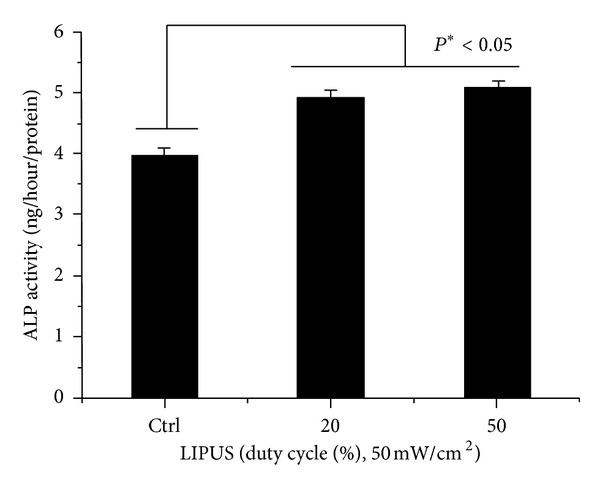
ALP activity cultured in different types of hABMSCs stimulated with LIPUS for 1 week. LIPUS groups exposed during 10 min/day at 20% or 50% duty cycle were significantly different (*P < 0.05) among groups (*n* = 3).

**Figure 8:**
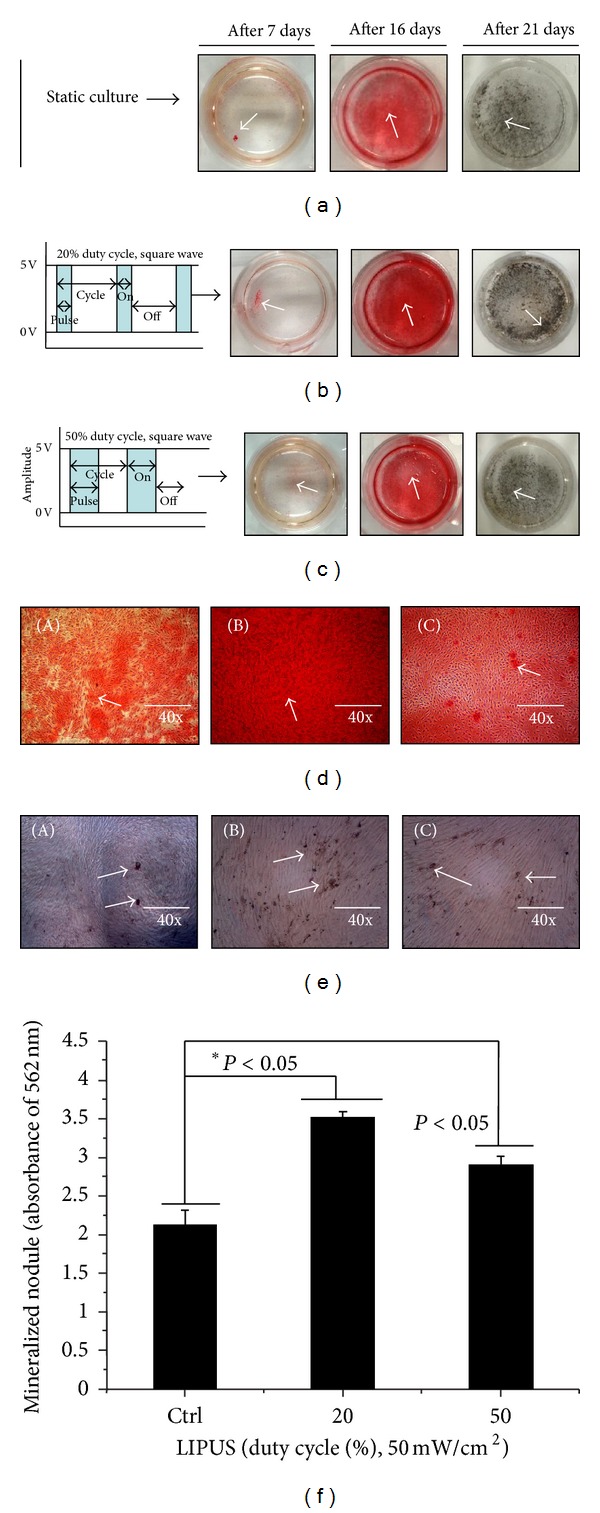
Representative images of hABMSCs after Alizarin red and von Kossa staining treatment in static condition (a), at 20% duty cycle (b), and at 50% duty cycle under LIPUS treatment (c) at 2-3 weeks after the addition of osteogenic differentiation media. Representative optical microscopic images of hABMSCs after Alizarin red staining treatment in static condition (A), at 20% duty cycle under LIPUS (B), and at 50% duty cycle under LIPUS (C) at 2-3 weeks were indicated (d). Mineralization images of von Kossa staining are shown in Figure 8(e). The short-pulsed duty cycle group was interestingly increased compared to the longer duty cycle group. Representative images were shown in Figures 8(d) and 8(e).  Figure 8(f) shows the optical density value of a mineralized nodule (absorbance of 562 nm) measured after destaining treatment.

We investigated the ALP activity of hABMSCs stimulated with LIPUS for 7 days. LIPUS groups exposed during 10 min/day at 20% or 50% duty cycle showed significant differences between groups (Figure 7). An early osteoblastic marker has relevance to the gene expression of other osteoblastic differentiation markers. Figures 8(a)–8(c) show representative images of hABMSCs after Alizarin red and von Kossa staining treatment in static condition (a), at 20% duty cycle under LIPUS (b), and at 50% duty cycle under LIPUS (c) at 2-3 weeks after the addition of differentiation media. Staining in the LIPUS group with 20% duty cycle was much more intense compared to the control, while the 50% duty cycle group was a bit generated.  Figure 8(d) shows representative optical microscopic images of hABMSCs after Alizarin red staining of cells in static condition (a) at 20% duty cycle under LIPUS (b) and at 50% duty cycle under LIPUS (c) at 2-3 weeks. Staining in the LIPUS group at 20% duty cycle was more intense than the static group. hABMSCs cultured with LIPUS under conditioned media showed increased calcium contents. Mineralization of von Kossa staining is shown in Figure 8(e). The short-pulsed duty cycle group was interestingly increased compared to the longer duty cycle group. Representative images ware shown in Figures 8(d) and 8(e). The LIPUS group exposed at 20% duty cycle was significantly different (*P < 0.05) than either of the other groups. This result shows that optimal LIPUS with the proper intensity, duty cycle, and time could enhance the *in vitro* growth and osteogenic differentiation of hABMSCs. 

### 3.6. Fluorescence Microscopy Analysis

**Figure 9:**
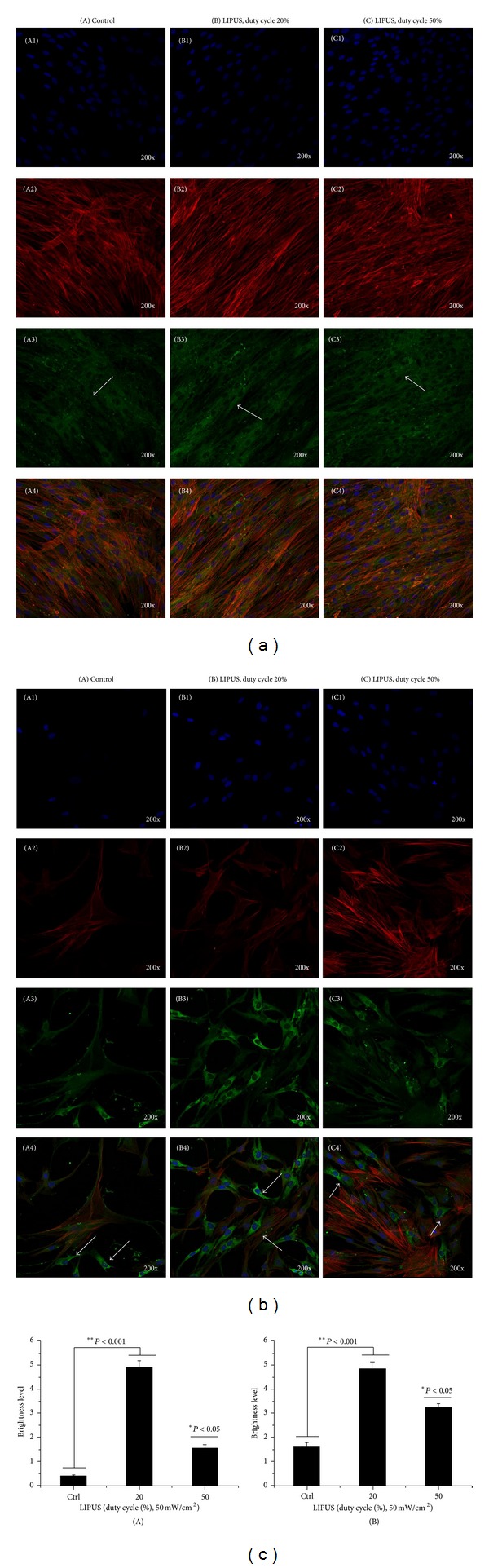
(a) Representative confocal laser microscopy images of hABMSCs cultured for 7 days in static conditions (A) or LIPUS induction at 20% duty cycle (B) or 50% duty cycle (C) after the addition of differentiation media: cell nuclei (blue), actin filaments (red), vinculin (green), and merged images of the fluorescence stains. Confocal laser microscopy images showed more intense observation in the LIPUS group compared to the control group. (b) Representative confocal laser microscopy images of hABMSCs cultured for 7 days in static conditions (A) or LIPUS induction at 20% duty cycle (B), or 50% duty cycle (C) after the addition of differentiation media: cell nuclei (blue), actin filaments (red), osteocalcin (green), and merged images of the fluorescence stains. Confocal laser microscopy images showed more intense observation in the LIPUS group compared to the control group. (c) Brightness level of vinculin (A) and osteocalcin (B) in florescence cell image treated by LIPUS induction.

Representative optical fluorescence microscopy images (Figure 9(a)) of hABMSCs cultured for 7 days in static conditions (A) or LIPUS induction at 20% duty cycle (B) or 50% duty cycle (C) after the addition of differentiation media: cell nuclei, actin filaments, osteocalcin and merged images of the fluorescence stains. Fluorescence images showed more lining-up observation at stimulation groups compared to control group (arrows: cell motion). Actin and vinculin were imaged to investigate possible rearrangements, reorientations, or both of cytoskeleton elements in hABMSCs exposed to LIPUS. Overall, actin microfilaments and vinculin intermediate filament structures were somewhat changed under LIPUS treatment.  Figure 9(b) shows representative confocal laser microscopy images of hABMSCs cultured for 7 days in static conditions (A) or LIPUS induction at 20% duty cycle (B) or 50% duty cycle (C) after the addition of differentiation media: cell nuclei, actin filaments, osteocalcin, and merged images of the fluorescence stains. Confocal laser microscopy images showed more intense observations in the LIPUS induction groups compared to the control group. Signal transduction via LIPUS ultimately could enhance adhesion molecules and then finally enhance osteogenesis. The results suggest that the LIPUS enhances the osteogenic differentiation and maturation of hABMSCs.  Figure 9(c) indicates relationship histogram of brightness level in florescence cell image treated by LIPUS induction. According to histogram of brightness, we could ascertain that LIPUS treatment had more intense rather than that of control.

## 4. Discussion

In this study, we investigated *in vitro *effects of LIPUS on the growth and osteogenic differentiation of hABMSCs for tooth tissue engineering. In particular, we found out effects of duty cycle of ultrasound, which could be delivered to tissues. Therefore, LIPUS which was 20% and 50% duty cycles during 10 min per day was induced. 

LIPUS is a form of physical energy that can be delivered into living tissue as acoustic intensity waves. Radical changes in density inherent in a healing tissue may well establish the gradients of physical strain [14]. Further, absorption of the ultrasound signal also results in energy conversion to heat [15, 16]. Though this thermal effect is extremely small for low frequency ultrasonic waves, well below 1°C, some enzymes, such as matrix metalloproteinase-1 or collagenase, are exquisitely sensitive to small variations of temperature [15–17]. Therefore, ultrasound may serve to e-establish or normalize effective metabolic temperatures in tissue-healing regions [15, 18]. Furthermore, incident radiation energy will be reflected at interfaces of distinct densities, resulting in complex gradients of acoustic pressure through the tissue [19]. The physical force produced by these intensity waves in living tissue can result in chemical events at the cellular level [20–22]. This may be generated through several possible mechanisms. The compression of microbubbles and acoustic streaming could have a direct effect on cell membrane permeability [23, 24]. Moreover, physical pressure exposed to LIPUS at the cell surface affected activation of cation channels [25]. The LIPUS also may influence the attachment of the cytoskeleton to the extracellular matrix [26]. In our study, actin and vinculin were captured to find out possible rearrangements, reorientations, or both of cytoskeleton elements in hABMSCs exposed to LIPUS. Namely, Actin microfilaments and vinculin intermediate filament structures were rearranged under LIPUS treatment. 

In this report, we evaluated whether LIPUS exposure with various duty cycles initiates osteogenic differentiation in hABMSCs. Physical force serves as an extracellular signal to a variety of cells, including bone cells. Several researchers have found an increase in cellular proliferation [27–30], and the production of prostaglandin E2 [31, 32] after invocations of various types of biophysical stimulation of bone cells. Several studies have showed that ultrasound stimulation leads to enhancement in protein synthesis [33, 34] and collagen synthesis [35]. *In vitro *studies have demonstrated increased chondrogenesis by increased aggrecan expression [36] after treatment with LIPUS. Wang et al. [37] showed that ultrasound stimulation led to increased vascular endothelial growth factor mRNA and protein levels in human osteoblast cells. Our result indicated that LIPUS increased osteogenic differentiation as well as proliferation and migration of hABMSCs, which is consistent with previous studies. Interestingly, migration and osteogenic differentiation were influenced by change of LIPUS duty cycle. The finding that duty cycle can influence proliferation and differentiation was not reported yet. Hence, as a future work, combining a variety of duty cycle and duration can be meaningful.

Dental implants are extremely useful for the restoration of oral function, including mastication, as well as for the aesthetic improvement in patients with tooth loss [38]. However, the success rate of implant is relatively low for the patients with poor quantity and quality of alveolar bone and with some diseases like osteoporosis. In the meantime, there is also an increasing need for shorter rehabilitation time in order to alleviate the inconvenience for patients [39]. Therefore, seeking an easy and effective method to improve and enhance the osseointegration of dental implants is necessary for dental clinicians and researchers. Some researchers have demonstrated that ultrasound stimulation increased surface expression of integrins in osteoblasts and that long-term stimulation also enhanced osteoblastic differentiation and inhibited osteoclastogenesis [40]. One study indicates that the cell population was increased significantly when osteoblasts were treated with ultrasound [41]. In the same manner, our study showed that ultrasound stimulation can enhance proliferation, migration, and differentiation of hABMSCs. In our previous study, proper ultrasound stimulation enhanced the proliferation of hABMSCs [13]. Yet, the study did not show how ultrasound stimulation affected on the migration and differentiation of hABMSCs. Hence, this study can be beneficial to dental regeneration.

The underlying mechanism of the mechanotransduction pathway involved in cellular responses to LIPUS is largely unknown. It has been demonstrated that LIPUS exposure increased cyclooxygenase-2 mRNA expression which leads to an increase in PGE2 (prostaglandin E2) and plays an essential role in the osseointegration of dental implants [42–44]. Another research showed that extremely low frequency pulsed electromagnetic fields (ELF-PEMFs) could enhance early cell proliferation in hABMSCs-mediated osteogenesis and accelerate the osteogenesis [45]. VEGF is a key regulator for angiogenesis which is essential to fracture healing [46]. Even though its concrete signals pathway is complicated and remains to be thoroughly understood, the present studies indicate that LIPUS has a favorable influence on osteoblasts.

It is foreseeable that miniaturized ultrasonic transducers may have the potential to improve patients' therapeutic experience. By using the treatment of LIPUS, the rehabilitation time may be shortened due to the acceleration of bone tissue regeneration. At the same time, the osseointegration can be strengthened, and a higher survival rate of the implants will ensue [47]. This indicated that optimal LIPUS device or stimulator with the proper intensity, duty cycle, and time could enhance the *in vitro* growth and osteogenic differentiation of dental stem cells for tooth tissue engineering.

## 5. Conclusions

The objective of this study was to find out the effects of LIPUS on proliferation and osteogenic differentiation of hABMSCs, which were treated with an intensity of 50 mW/cm^2^ and exposure time of 10 min/day. Pulsed ultrasound (1 MHz) at duty cycles of 20% and 50% was used in this study. The results are as follows: hABMSCs exposed at duty cycles of 20 and 50% had similar cell viability, which was higher than that of control. The mineralized nodule formation was markedly increased after LIPUS treatment than that of control group. Gene expression indicated that LIPUS treatment had a positive influence on the expression of mRNA for ALP and Col-I. Our study demonstrated that hABMSCs undergoing LIPUS could be positively influenced toward osteogenic differentiation. Osteoinduction of osteocalcin showed more intense observations for the LIPUS induction groups compared to the control. Signal transduction via LIPUS ultimately could enhance adhesion molecules, and then generate osteogenesis. These results suggest that LIPUS treatment could affect the cell viability and osteogenic differentiation of hABMSCs, as well as be part of effective treatment methods for clinical applications.

## Supplementary Material

Migration Effects of LIPUS via existence and nonexistence of FBS or Migration Effects of LIPUS with Media Component Variation.

## Figures and Tables

**Table 1:** Primers used for RT-PCR.

Gene	Accession number	Primer sequence	Predicted size (bp)
CD29	NM_002211	5′-AATGAAGGGCGTGTTGGTAG-3′ 5′-CGTTGCTGGCTTCACAAGTA-3′	337
CD44	X55938	5′-ACCGACCTTCCCACTTCACAG-3′ 5′-GCACTACACCCCAATCTTCAT-3′	168–200
Col-I	NM_000088	5′-CTGGCAAAGAAGGCGGCAAA-3′ 5′-CTCACCACGATCACCCACTCT-3′	503
OCN	X53698.1	5′-CATGAGAGCCCTCACACTC-3′ 5′-AGAGCGACACCCTAGACCG-3′	315
GAPDH	AF017079	5′-GGGCATGAACCATGAGAAGT-3′ 5′-CCCCAGCATCAAAGGTAGAA-3′	497
