# Supplementary figures and images for: In Vitro Effects of Low-Intensity Pulsed Ultrasound Stimulation on the Osteogenic Differentiation of Human Alveolar Bone-Derived Mesenchymal Stem Cells for Tooth Tissue Engineering
Source: Biomed Res Int. 2013 Sep 30;2013:269724. doi: 10.1155/2013/269724 (PMC3806253; doi:10.1155/2013/269724)

# Supporting Information, S1

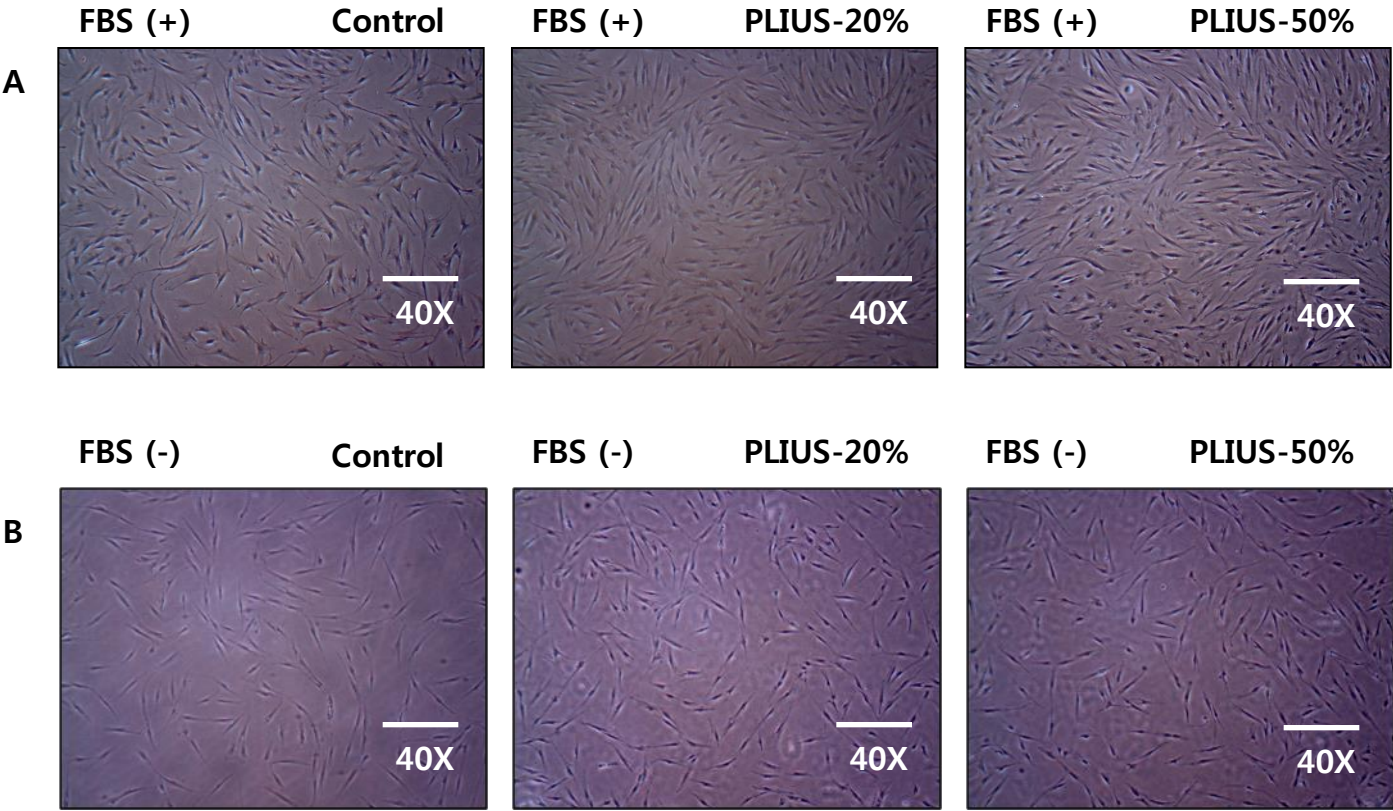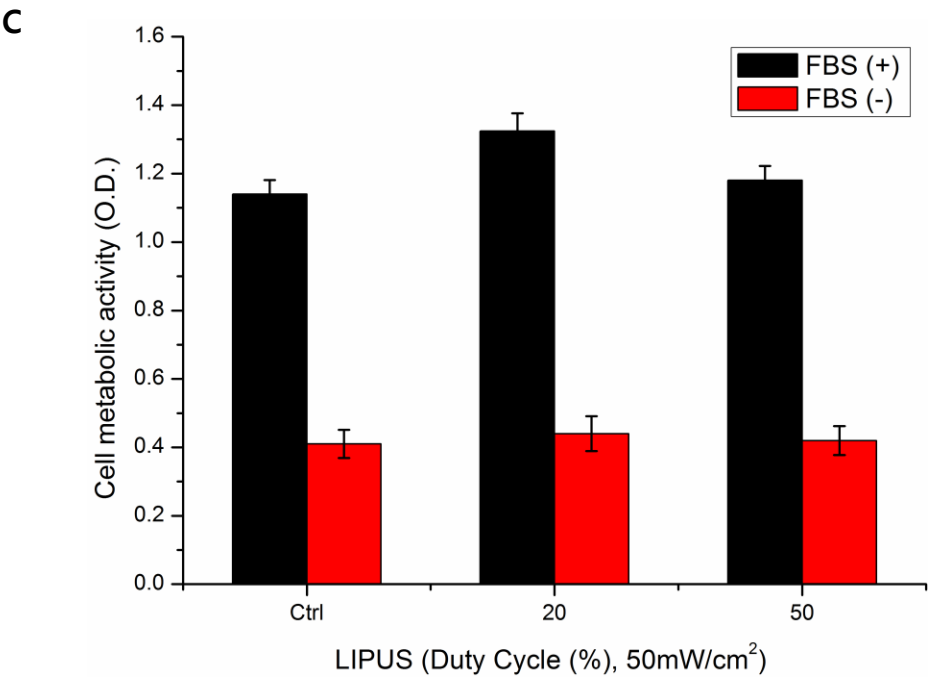

Supporting Information, S2

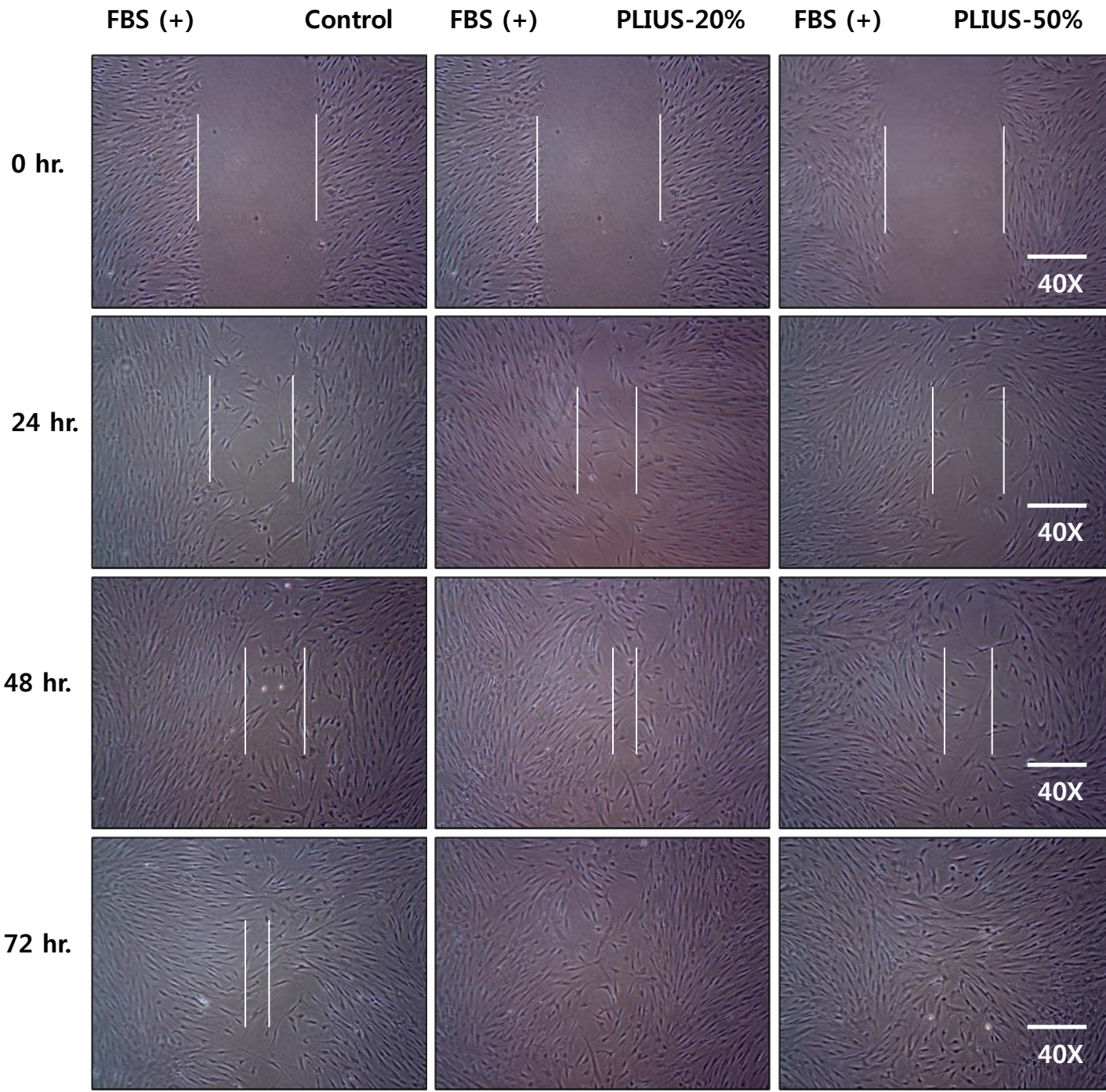

Supporting Information, S3

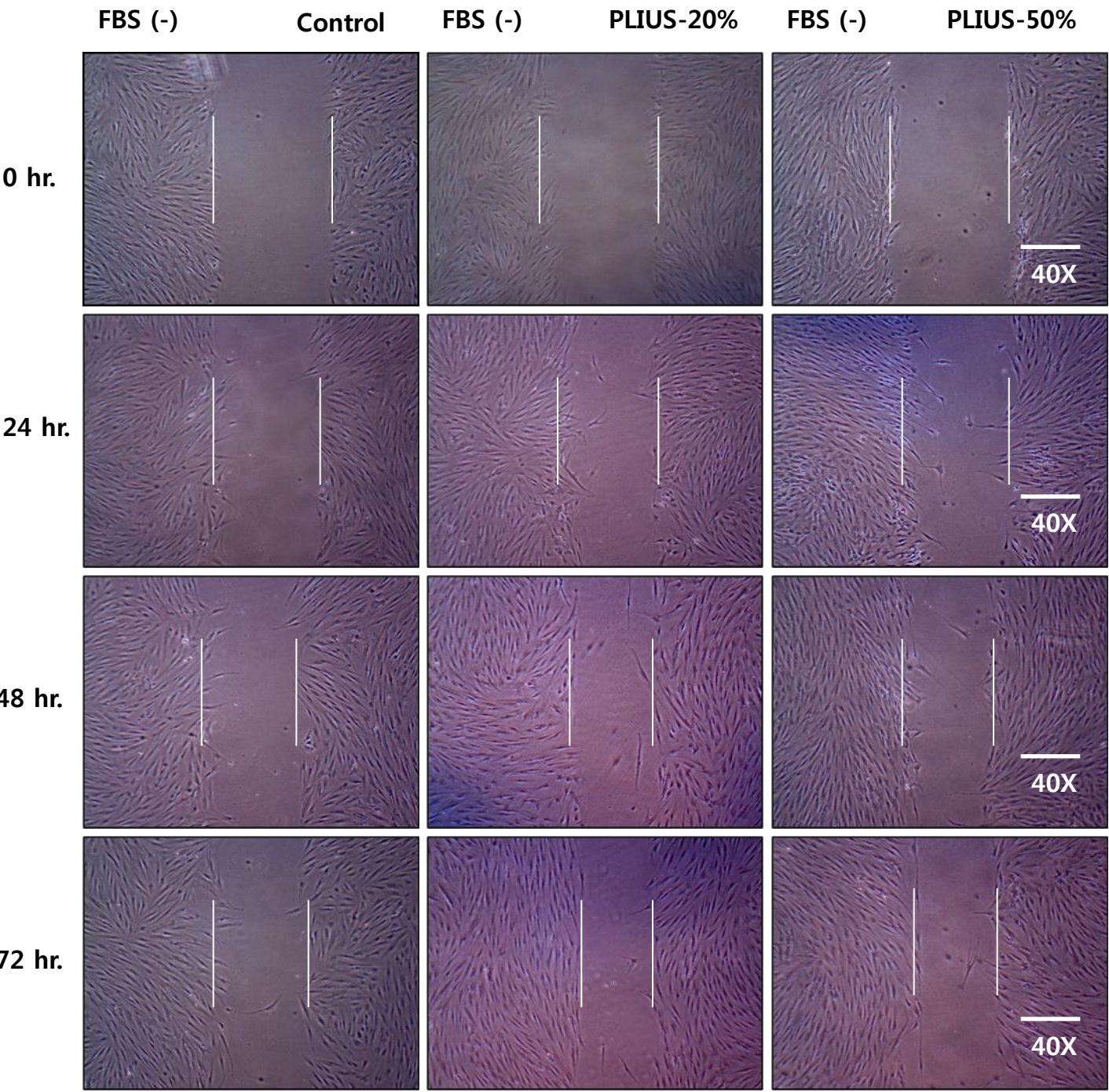

Supplement: Supplementary file 1 — Migration Effects of LIPUS via existence and nonexistence of FBS or Migration Effects of LIPUS with Media Component Variation. [file 269724.f1.pdf]
